# Hypermentalizing in Social Anxiety: Evidence for a Context-Dependent Relationship

**DOI:** 10.3389/fpsyg.2019.01501

**Published:** 2019-07-09

**Authors:** Sergi Ballespí, Jaume Vives, Carla Sharp, Andrea Tobar, Neus Barrantes-Vidal

**Affiliations:** ^1^Department of Clinical and Health Psychology, Universitat Autònoma de Barcelona, Barcelona, Spain; ^2^Department of Psychobiology and Methodology of Health Sciences, Universitat Autònoma de Barcelona, Barcelona, Spain; ^3^Department of Psychology, University of Houston, Houston, TX, United States; ^4^Department of Mental Health, Fundació Sanitària Sant Pere Claver, Barcelona, Spain; ^5^Centre for Biomedical Research Network on Mental Health (CIBERSAM), Instituto de Salud Carlos III, Madrid, Spain

**Keywords:** social anxiety, social cognition, mentalization, hypermentalizing, context-dependency, self-image, self-esteem

## Abstract

Social anxiety (SA) means fear of scrutiny and of others’ negative evaluation, thus indicating that hypermentalizing (HMZ) (i.e., the over-attribution of intentions and thoughts to others) might be the most common error of social cognition in SA. However, evidence for this is weak. One explanation is that HMZ is not stable in SA, but rather context-dependent. The first aim of the current study was testing this hypothesis. The second aim was analyzing whether the association between SA and HMZ is moderated by a negative self-image. One-hundred and thirteen young adults (85.8% females; *M* = 21.1 years old; SD = 2.7) were assessed on measures of SA, HMZ, and self-image. Given the over-representation of females, conclusions may not be safely extrapolated to males. Results revealed that HMZ is associated with SA only in the self-referential social situation [*B* = 2.68 (95% CI: 0.72–4.65), *p* = 0.007]. This supports that HMZ is not global in SA (i.e., a stable cognitive style), but rather is active only in some contexts. Implications for the conceptualization and treatment of SA are discussed. Contrary to predictions, neither self-esteem, nor positive or negative self-schema moderated the association between SA and self-referential HMZ. This contradicts findings in the field of paranoid delusion and requires replication, including measures of implicit self-esteem.

## Introduction

Social Anxiety Disorder (SAD) is characterized by marked fear of scrutiny and negative social evaluation (American Psychiatric Association [APA], 2013). This disorder represents the clinical extreme of the Social Anxiety (SA) Spectrum (Schneier et al., 2002), affects 2 to 7% of adults annually, and is frequently associated with depression (Beesdo et al., 2007). Despite the highly impairing and chronic nature of SAD, only half of people with the disorder ever seek treatment and most of them do so after 15–20 years of experiencing symptoms (American Psychiatric Association [APA], 2013).

The challenge in recovery from SAD highlights the importance of preventing clinical levels of SA, and emphasizes the need to identify some of the underlying mechanisms along the SA Spectrum (Schneier et al., 2002). Given the acknowledged importance of cognitive aspects in SA, the role of attention and memory biases has been widely analyzed (Heeren et al., 2015). However, little is known about the role of social cognition, especially regarding aspects such as social perception and mentalizing (Plana et al., 2014).

Mentalizing (MZ) is defined as the higher-order cognition that allows the identification of mental states (intentions, desires, feelings) that underpin human behavior (Fonagy and Bateman, 2016). MZ is a multidimensional process (Choi-Kain and Gunderson, 2008; Kim, 2015), usually referred to as social cognition or Theory of Mind (ToM), the latter specifically referring to the capacity to infer or imagine what is happening in another’s mind. This process is hypothesized to play a role in SA Spectrum because at the core of SA is the fear of negative evaluation, meaning that people with SA may tend to avoid social interaction because of the frightening mental states that they attribute to other people (i.e., the intention of scrutiny and negative evaluation). Put differently, the interpretations of what others think or intend are the core of fear in people with SA.

While some classic ToM tasks have been employed in children with SA (Banerjee, 2008), a relatively new approach to studying MZ involves the assessment of excessive ToM (Dziobek et al., 2006), or hypermentalizing (HMZ) (Sharp et al., 2011). HMZ refers to the over-attribution of mental states to others (such as the intention of negative evaluation), that goes far beyond the relevant context. The concept of HMZ was originally associated with paranoid ideation (Langdon and Coltheart, 1999), and has been found in Borderline Personality Disorder (BPD; Sharp et al., 2013).

The essential component of SA (American Psychiatric Association [APA], 2013), suits the definition of HMZ because the fear to scrutiny is excessive since it is unlikely or paranoid (without basis in context), and the individual is convinced in his/her attribution of negative evaluation rather merely considering it possible. This makes people with SA exhibit MZ reasoning more similar to those in the paranoid spectrum (over-attributing intentions to others), than those in the autism spectrum (Baron-Cohen et al., 1985). “Excessive theory of mind” or HMZ should be the most common error of MZ in individuals with SA. This is consistent with the interpretation bias found by most studies about cognition (Clark and McManus, 2002; Miers et al., 2008).

Cognition is an important element in SA, and distortions in attention, interpretation, and memory biases have been thoroughly analyzed (Heinrichs and Hofmann, 2001). The interpretation bias is one of the most extensively analyzed from the information processing paradigm and refers to the tendency to misinterpret the social scenario by exaggerating cost estimations of negative outcome, or the negative consequences of the social situation (Chen et al., 2018).

However, despite the social dimension of SA, fewer studies analyzed *social cognition*. To analyze SA from social cognition implies an additional step as it moves the interpretation bias from the scenario (i.e., the interpretation of the situation as a potential threat, or the excessive attention to negative consequences), to the mental states (i.e., the interpretation or attribution of intentions, feelings and judgments to others).

Interestingly, among the very few studies that analyzed social cognition errors in individuals with SA, only two of them found an association between HMZ and SA. Hezel and McNally (2014) referenced greater prevalence of “excessive ToM” in people with SAD compared with non-SAD individuals, albeit demonstrating a small effect size. Washburn et al. (2016) reported higher rates of “excessive ToM” in SAD groups (i.e., SAD and SAD+Depression) compared to people with major depression, but not compared to controls. Thus, against theoretical predictions, evidence is limited and implies a modest association between HMZ and SA. A possible explanation is that HMZ is not a general and stable attribute of SA.

In disorders like BPD, HMZ is the underlying social-cognitive problem (Sharp et al., 2013), and is attributed to absence of proper attachment relationships in childhood (Fonagy and Bateman, 2008; Fonagy and Luyten, 2009; Fonagy et al., 2011), which are necessary for the correct development of MZ capacity (Fonagy and Target, 1997). However, there is no reason to suppose that MZ problems in SA are similarly rooted in disrupted attachment relationships in childhood.

Etiological models of SA, as in Rapee and Spence (2004), have established that SA is heavily influenced by genetic risk factors (with heritability estimates around 0.65), which can be expressed as a highly reactive or an inhibited temperament. Such temperaments make children more susceptible to environmental influences – such as modeling by anxious parents (American Psychiatric Association [APA], 2013). Thus, although early social environment plays a role in moderating genetic dispositions (Nachmias et al., 1996), attachment-related trauma is not usually underlying the development of SA as it is in BPD. Therefore, it is possible that most people with SA had good enough attachment relationships and developed normal or even improved MZ capacity (Mink et al., 2014), at least in non-distressing social situations (e.g., those with close friends or family).

In contrast, since arousal affects MZ capacity, as noted by Fonagy and Bateman (2016), Frith and Frith (2003), and Luyten and Fonagy (2015), it is possible that those situations that become distressing to people with SA – i.e. uncertain social situations in which they feel involved – elicit MZ errors because they activate self-referential MZ. This may partly explain why prior studies were unable to show that general HMZ accounted for most of the MZ errors associated with SA.

The aim of the current study was to evaluate this hypothesis. It is assumed that if HMZ was generalized in people with SA, it should appear in all MZ activity, even in non-frightening social situations. By contrast, if HMZ in SA is context-dependent, then it should be especially present in frightening social situations, operationalized here as uncertain situations that involve the individual and therefore foster self-referential MZ.

Thus, we hypothesize that non-self-referential HMZ – measured with a computerized procedure in which participants mentalize about the characters of a film – will be less strongly associated to SA than self-referential HMZ, as measured through a social situation that directly involves the participant.

To analyze this hypothesis in a sample from general population, we adopt the criterion of the Harvard’s group (Kagan et al., 1987) to operationalize as “presence” of SA those cases with scores above percentile 85th in measures of SA (Kagan et al., 1998). This criterion is psycho-physiologically based (Kagan et al., 1988) and widely used in literature about SA spectrum (see Ballespí et al., 2013 for a review).

Assuming support for our hypothesis, we additionally aimed to explore why those with SA tend to HMZ in self-referential social situations. Clark and McManus (2002) suggest that SA is maintained through a vicious cycle in which people engage in a series of biased pre- and post-interaction cognitive processes that act to generate anxious emotion and impair social performance. From this point of view, context-dependent HMZ may appear as an intermediate process between trait-SA (i.e., tendency to SA), and symptoms (expression of this disposition). However, it is still unknown what leads people with SA to project the intention of a negative judgment onto others – a judgment which leads to fear and social avoidance. Similar to those with SA, individuals with persecutory ideation are by definition frequently misreading the intentions of other people and over attributing mental states to others (i.e., HMZ).

In the field of psychosis, self-image has been identified as a psychological mechanism associated with persecutory beliefs and attributional biases (Valiente et al., 2011, 2014). Bentall and colleagues’ “defensive model” (see Thewissen et al., 2008; Udachina et al., 2009; Melo and Bentall, 2013) of paranoia establishes that those with persecutory ideation attribute to others the responsibility, actions, and intentions of negative events in order to protect the self from low self-esteem and negative self-beliefs. Freeman and Garety’s investigations (Freeman et al., 1998; Smith et al., 2006; Freeman, 2007), also show that low mood, low self-esteem, and negative schematic beliefs contribute to the development of psychotic symptoms but, in this case, in a direct and non-defensive way. In contrast to Bentall’s suggestion, these authors suggest that low self-esteem in paranoia may not depend on psychotic, but on neurotic, processes (Freeman and Garety, 2003).

Beyond differences, both the aforementioned models support the idea that self-concept and self-esteem play an important role in the development of psychotic delusion (Bentall et al., 2001; Freeman, 2007). The excessive, “paranoid” fear to others’ scrutiny and negative evaluation can be considered a lower-level delusion from a dimensional point of view, since the mental states attributed to others tend to be held as true instead of only possible. Consequently, it is also possible that self-image may play a role in the development of “neurotic” over-attributions (i.e., the suspicion of scrutiny and negative evaluation), that people with SA do when HMZ.

Thus, our second aim was to evaluate if self-referential HMZ depends on low self-esteem. Specifically, we hypothesized that poor self-image (i.e., low self-esteem or negative self-schema), may moderate the association between SA and self-referential HMZ, because a negative idea of one’s self might lead people to fear that others judge them as they do; that is, to fear that others consider (or “discover”), they are as they believe themselves to be (i.e., according to a poor self-image: “bad” or defective in some way). This hypothesis assumes that people with SA tend to HMZ because they assume as certain what they fear: that others will consider them in the same way they do. The over-attribution to others of one’s own mental state (i.e., negative self-judgment), not only suits the definition of HMZ but it also suggests that HMZ in SA arises from negative self-image. According to the literature (Smith et al., 2006; Kesting and Lincoln, 2013) both affective and cognitive dimensions of self-image were considered, so the moderating role of self-esteem and self-concept was analyzed.

## Materials and Methods

### Participants

**FIGURE 1:**
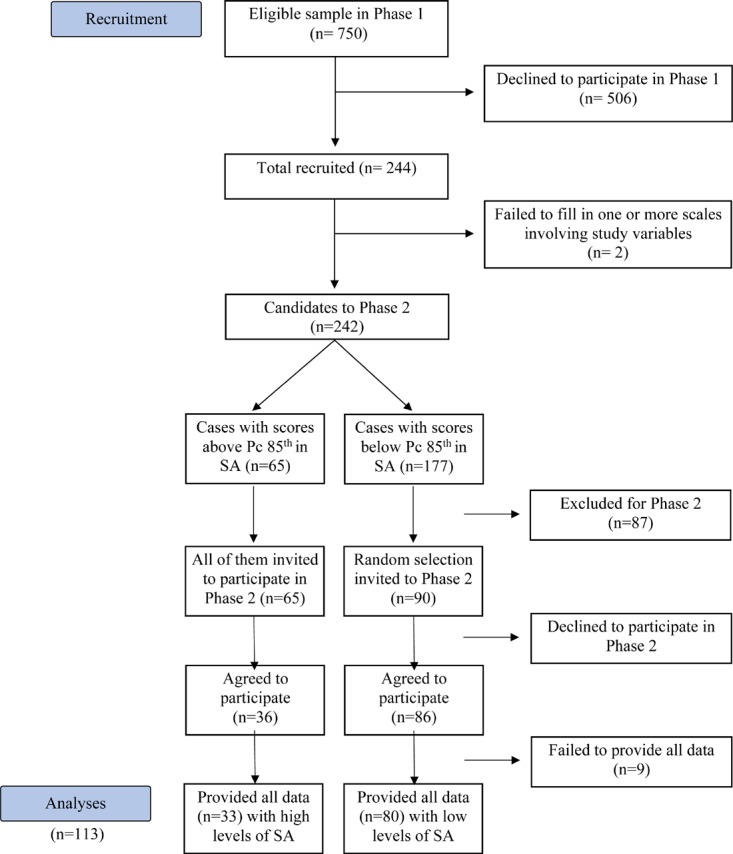
Sample recruitment flow chart.

Participants included 113 individuals (97 females, or 85.8%; 85% White-European, 14% Latino, 1% Arabic) with a mean age of 21.1 years old (SD = 2.7), who participated in the Phase 2 of a study about MZ and SA. In the Phase 1 of this study, the students of 1st and 2nd year of Psychology Degree were encouraged to report levels of SA through the SPAI-B (Garcia-Lopez et al., 2008; Piqueras et al., 2012; see Section “Instruments”) and the Brief Version of the Fear of Negative Evaluation Scale (BFNE) (Leary, 1983; see also Gallego et al., 2007). To operationalize “presence” of SA basing on a spectrum perspective (Schneier et al., 2002), the criterion of the Harvard’s group was used (Kagan et al., 1987, 1988). This criterion considers that the extremes of 15% in a distribution of SA are qualitatively different than middle-levels positions (Kagan et al., 1998). However, in contrast to classic studies which compare two extreme groups (those with scores above percentile 85th in a SA measure, with those below percentile 15th, i.e.: the extremes of 15% of the distribution of that measure), in order to avoid the extremes discontinuity, we aimed to compare those with SA (i.e., cases above percentile 85th in measures of SA) with a random selection of those below percentile 85% (see Figure 1).

Thus, from the approximately 750 students invited to study, 244 provided written informed consent and completed the screening measures. From those 244, the 65 participants with high levels of SA in the screening measures (i.e., scores above the 85th percentile in the SPAI-B or in the BFNE), along with a randomized sample of 90 of the 177 “controls” (i.e., those with SA scores below the 85th percentile in SPAI-B and BFNE) were invited to participate in Phase 2. From the 155 potential participants for Phase 2, 122 agreed to participate and 113, or 73%, provided written informed consent for this phase of the study and came to the lab to complete additional measures. The 42 individuals who did not participate were those who did not answer the phone calls from the research team (10 participants), and those who cited not being interested or having logistic difficulties (i.e., conflicts with work schedule, distance to the lab). It is of course possible that SA could be a reason for some participants to avoid this part of the project with “in-person” assessments. The final sample was composed of 80 participants with low levels of SA, and 33 participants considered to have high level SA (i.e., with scores above the 85th percentile on the BFNE or the SPAI-B).

### Measures

#### Social Anxiety

According to literature, two of the most widely used instruments for the screening of SA were used in phase 1 to select people with high (i.e., scores > 85th percentile on at least in one measure), and low (i.e., scores < 85th percentile in both measures), for phase 2. The SPAI-B (Garcia-Lopez et al., 2008) was selected because it emphasizes the measuring of psychophysiological signs of SA and of social avoidance. The BFNE (Leary, 1983) was selected to emphasize the cognitive component of SA. Both instruments are described below.

##### Social phobia and anxiety inventory – brief form (SPAI-B) (Garcia-Lopez et al., 2008; Piqueras et al., 2012)

This is a cost-effective 16-item version of the SPAI (Turner et al., 1989), particularly useful as a screening measure of the three-response system approach (i.e., motor, psychophysiological and cognitive), for SA, and consistent with the original scale. There is evidence for construct and concurrent validity both in samples of adolescents and adults, and it possesses good test–retest reliability (*r* = 0.60 in 6 months), and excellent internal consistency (Cronbach’s α = 0.92). The internal consistency in the current sample is α = 0.93.

##### Brief Version of the Fear of Negative Evaluation Scale (Leary, 1983)

This scale consists of 12 items scored on a 5-point Likert scale and it assesses a defining characteristic of SA and SAD: the degree of fear to negative evaluation (American Psychiatric Association [APA], 2013). There is evidence for convergent and concurrent validity of this scale, and it shows an excellent internal consistency (Cronbach’s α = 0.90), both in the original Spanish version (Gallego et al., 2007; Pitarch, 2010) and in the current study.

#### Hypermentalizing

In order to test if this MZ error is context-dependent in SA, we obtained a measure of HMZ in two different contexts. First, we used the movie for the assessment of social cognition (MASC) (Dziobek et al., 2006), described below. To score the MASC, participants have to think about the mental states of the characters of a film. Given that participants are not personally involved in the social situation, and they individually score the MASC alone in a lab, HMZ errors will not be influenced by SA in this case; thus, we consider that the MASC scale of HMZ is a good operationalization of non-self-referential HMZ. By contrast, in our second procedure [the method for inducing mentalization in a self-referential situation (MIMS), see below], we expose participants to a social situation in which they are involved, so their MZ activity will be self-referential. Given that, people prone to SA are expected to experience SA in this situation, because they have to talk about themselves with a non-familiar interviewer.

##### Movie for the assessment of social cognition (Dziobek et al., 2006)

This is a widely used and well-established method for the assessment of the ToM reasoning style. Administration of the MASC takes about 45 min. It requires that participants watch a 15 min film about four characters getting together for a dinner party. The video is paused 46 times and multiple-choice questions concerning characters’ feelings, thoughts, and intentions are asked. The participants answer the questions that follow every one of the 46 video segments by choosing one of the four options of response regarding the mental states of the characters. In all cases, four response options are offered: adequate MZ, hypomentalizing (under-mentalizing), no MZ, and HMZ. The MASC provides a score for every one of these four scales. The Spanish version of the MASC (Lahera et al., 2014) shows psychometric properties as good as those of the original version, including good internal consistency (α = 0.86). The computerized administration of the measure (as it only provides final scores), does not allow estimation of the internal consistency in the current sample.

##### Method for inducing mentalization in a self-referential situation

This method was created to assess MZ through a real social self-referential situation. It is inspired by the MASC (Dziobek et al., 2006; see the description above), in the sense that participants are asked about different moments of a social interaction. However, in the MIMS the social situation is not fictitious like in the MASC (i.e., a movie watched in a computer), but it is real and involves the participant (i.e., it consists of a standardized interview of the participant). In this context, the mental states about which the participants will be asked are not those of characters, but those of the interviewer, a person with whom participants are interacting. The administration of the MIMS includes three steps (before, during, and after the interview).

###### Before the interview

Participants were informed that after a sequence of lab situations, such as Rosenberg’s Self-Esteem Scale (RSES) (Rosenberg, 1965), a MZ interview, and the MASC, they would finish with an important and definitive interview.

###### The interview

The content of the interview consisted of personal questions, inspired by the initial questions of the SCID II (First et al., 1997, 1999; i.e., *How would you describe yourself? How do you think that others would describe you? What kinds of things have you done that other people found disgusting or annoying? If you could change your personality, how would you like to be different?*), but also including other questions (i.e., *What do you want to do in life?* or *If you met a genie who granted you two wishes, what would you ask for?*). It was expected that personal questions would help to increase arousal especially in people with SA. Neither the actual questions nor the answers were important for the purpose of the assessment. The focal point was the standardized sequence of “neutral social signs” that the interviewer introduced in the interview in order to activate MZ in the participants. For instance, after the instruction (i.e., *“Now I am going to ask you some questions about the kind of person that you are, that is, how you feel and behave in general”*), the first question is asked: “*How would you describe yourself?”* At the first pause in the participant’s response the interviewer *clears his/her throat* (item 1). If the participant takes more than 5 s to resume answering, the interviewer clears his/her throat again. When the participant finishes, the interviewer again clears his/her throat and simulates writing down the answer. Then the interviewer asks the second question: *How do you think that others would describe you?* When participants are carrying on their response the interviewer *yawns* (item 2). When participants are asked about their defects and virtues, the interviewer hesitates and expresses surprise, respectively (items 3 and 4). And so on with other “neutral social signs” (e.g., to stare, to knit the brow, to be silent for a while, to hesitate before writing a response, to pretend to improvise a question out of the interview), that are expected not to be negatively interpreted by controls but indeed by SA participants.

###### After the interview

Immediately after the interview, participants were placed alone in a room with a computer in order to allow them to rate from 1 (“I do not agree at all”), to 7 (“Completely agree”), the different possible attributions of every “neutral social sign.” For instance, the first question was: “*During the interview, it is possible that the interviewer coughed or cleared his/her throat. Why do you think he/she did this?*,” and the four responses that the participant had to rate from 1 to 7 were: “*(a) He/she needed to clear his/her throat*” (Correct, because it is likely; refers to a specific attributed need); “*(b) I possibly said something wrong or negative and he/she tried to cloak his/her reaction*” (HMZ, because this attribution goes beyond the context); “*(c) It is probably a tic; some people have them*” (under-mentalizing, because it refers to a global tendency and tends to place the cause out of mind); “*(d) It is not worth wondering why people clear their throat*” (no MZ, because the response lack any reference to mental states in order to explain behavior).

So, this method makes it possible to ask participants about the mental states beyond the behavior of a person (the interviewer), with whom they have been interacting in a real (and self-referential) situation, instead of the characters of a video watched alone in a lab.

Principal Components Analysis revealed that the MIMS suited a 4-factor solution according to the scree-plot and criteria of parsimony, interpretability, and explained variance (40%; weights ranged from 0.3 to 0.8). Varimax rotation highlighted a first factor grouping items of HMZ versus other 3 factors grouping items of correct-, hypo- and no-MZ. Basing on the 6 items of HMZ that: (a) were more clearly referred to HMZ according to a peer-review process, and (b) unequivocally weighted only in the factor of HMZ (weights ranged 0.55–0.79), a total score of self-referential HMZ was calculated.

The score of self-referential HMZ was the sum of the scores of participants on those items, ranging from 6 to 42, and showed an acceptable internal consistency according to Cronbach’s α = 0.75. The HMZ scale of the MIMS was positively correlated with theoretically associated constructs, such as the scale of Fear to Negative Evaluation of the SASA (*r* = 0.31, *p* = 0.001), and with the HMZ scale of the MASC (*r* = 0.25, *p* = 0.009). This is evidence for convergent validity. Moreover, it was negatively correlated to Rosenberg’s Scale of Self-Esteem (*r* = –0.35, *p* = 0.002) and showed no association with the other scales of the MASC (correct-, hypo- and no-MZ), which is evidence for discriminant validity.

#### Self-Image

According to literature, measures of self-esteem (SE) and self-concept were used to assess the affective and the cognitive dimensions of self-image (Smith et al., 2006). The RSES (Rosenberg, 1965) is one of the most widely used instruments to assess self-esteem. It consists of 10 items rated through a 4-point Likert-type scale and provides a single global score of SE. The Spanish version shows good psychometric properties (Martín-Albo et al., 2007) and the internal consistency in the current sample was excellent (Cronbach’s α = 0.91). The Brief Core Schema Scales (BCSS) (Fowler et al., 2006) is a 24-item self-report that provides measures of self and others’ positive and negative schemas. The scales of positive and negative self-schema were used in the present study as an operationalization of self-concept. These scales showed excellent internal consistency (Cronbach’s α ≥ 0.90) in the current sample.

### Procedure

The study meets ethical standards according to Declaration of Helsinki and was approved by the Ethics Committee of the Universitat Autònoma de Barcelona (CEEAH, Spain; CEEAH no. 4061). We report how we determined and obtained our sample, all data exclusions, all manipulations, and all measures in the study. Data used in the analyses are provided as Supplementary Material. The study was funded by the Catalan Government (Support to Research Groups, 2014SGR1070) and by the Spanish Ministry of Economy and Competitiveness, PSI2014-56303-REDT and PSI2014-52962-P). The authors declare no conflict of interest.

The study consisted on 2 phases. In phase 1, participants rated several on-line questionnaires (including SPAI-B and BFNE) after providing written informed consent. These measures involved the SA-screening measures used in this study to design the groups with high and low SA. The design of the groups was based on the Harvard’s group criterion to operationalize “presence” of SA (Kagan et al., 1987, 1988, 1998). Following this criterion, among the 242 who agreed to participate and provided full data in Phase 1 (see Figure 1), those above percentile 85th in the SPAI-B or BFNE were selected as “cases with high SA” (*n* = 65), and a random selection of those below percentile 85th (90 of 177) were selected as cases with “low SA.” In Phase 2, after providing further written informed consent for this second phase, 33 participants with high SA and 80 participants with low SA took part in different laboratory tasks (necessary for other studies), including the measures here used: the MASC, the MIMS, and self-image measures Supplementary Material. The participation in the phase 2 was compensated with 20 euros.

### Statistical Analysis

*T*-test and χ^2^ (Fisher’s exact test was used in case of cells with expected count less than 5), were performed to test for statistical independence between exposure and demographic variables. Linear regressions were performed to investigate the association between SA and self- and non-self-referential HMZ, as well as to appraise the moderating role of self-esteem (measured with Rosenberg’s Scale), and self-concept (i.e., positive and negative self-schemas measured with BCSS), on the relation between SA and self-referential HMZ.

We conducted *post hoc* power analyses using G^∗^Power v.3.1.9 (Faul et al., 2007) for a small to medium effect (*f*^2^ = 0.1), α = 0.05, and the smallest sample size used in our analyses (*n* = 109), two explanatory variables, two control variables, the power (1 – β) was 0.84.

All data analyzes were using IBM SPSS Statistics v19.0 package (IBM Corp., 2010). Assumptions of independent errors (Durwin–Watson test), homoscedasticity (Plot of standardized predicted values against standardized residuals), normality of residuals (P–P plot), absence of multicollinearity (VIF and tolerance), and of influential cases (Cook’s distance), were checked. All models tested met the assumptions. The results of the association between anxiety (high/low) and each response variable, as well as the moderation effects are presented as linear regression coefficients (B), reporting 95% confidence intervals (95% CI), and *p*-values. Statistical significance was set at *p* < 0.05.

**Table 1:** Descriptive statistics of variables of the study.

	Low SA			High SA		
	*M* (95%CI)	Min	Max	*M* (95%CI)	Min	Max
1. Non-self-referential HMZ	3.64 (3.13–4.15)	0	9	4,37 (3.49–5.25)	1	11
2. Self-referential HMZ	12 (11.07–12.93)	6	22	14.10 (11.74–16.39)	6	30
3. Self-esteem	23.03 (21.95–24.10)	10	30	18.30 (16.07–20.53)	9	29
4. Negative self-schema	7.83 (5.94–9.72)	0	24	10.3 (7.45–13.15)	0	21
5. Positive self-schema	16.15 (15.25–17.06)	6	24	15.50 (14.08–16.92)	5	22

*Social anxiety (SA), 95% confidence intervals (95% CI), mean (M), minimum (Min), maximum (Max).*

## Results

### Demographic Characteristics and Descriptive Statistics of Study Variables

There were no statistical differences in the distribution of age [*t*(111) = 0.62, *p* = 0.54], sex (Fisher’s exact test, *p* = 0.39), ethnic group (Fisher’s exact test, *p* = 0.39), or socioeconomic status (Fisher’s exact test, *p* = 0.30) between the group with low level of SA (*N* = 80; 84% of females, 83% Caucasian, 16% Latino, 1% Arabic; aged *M* = 21.3 years old, SD = 2.9), and the group with high level of SA (*N* = 33; 91% of females, 91% Caucasian, 9% Latino; aged *M* = 20.8 years old, SD = 2.1). The overall distribution of socioeconomic status was: 8% Low, 33% Medium–Low, 23% Medium, 31% Medium–High, 5% High. Table 1 presents the descriptive statistics of the study variables for each one of the comparison groups.

### Effects of SA on Self- and Non-self-referential HMZ

To analyze the relationship between SA and HMZ, linear regression models were tested with the self- and non-self-referential HMZ as response variables. Final models show that the association between SA and self-referential HMZ was statistically significant [*B* = 2.68 (95% CI: 0.72–4.65), *p* = 0.007], but not the association with Non-self-referential HMZ [*B* = 0.58 (95% CI: –0.34–1.49), *p* = 0.22]. This indicates context-dependency in the relationship between SA and HMZ.

### Moderators of the Effect of SA on Self-Referential HMZ

To analyze the moderating role of self-image on the relationship between SA and HMZ, linear regression models were tested with self-referential HMZ as the response variable. Table 2 shows the final models where no moderation effect was found to be statistically significant. Therefore, the relationship between SA and Self-referential HMZ is not moderated by self-esteem, nor by self-concept (i.e., positive and negative self-schema).

## Discussion

The phenomenological definition of SA (American Psychiatric Association [APA], 2013) highlights the tendency to attribute intentions of scrutiny and certainty of negative judgments to others. This is an essential feature of SA. From the perspective of social cognition, this mechanism suits the definition of “excessive theory of mind” (Dziobek et al., 2006) or HMZ (Sharp et al., 2013), so it was reasonable to expect that people with SA will exhibit a pattern of social cognition more similar to that of people with paranoia (i.e., “*to make assumptions about other people’s mental states that go so far beyond observable data that the average observer will struggle to see how they are justified*”; Sharp et al., 2013, p. 4) than to that of people with autism spectrum disorders (i.e., deficit of ToM, hypo-mentalizing or mind-blindness; Baron-Cohen et al., 1985). However, previous studies did not find a clear relationship between SA and HMZ (Hezel and McNally, 2014; Washburn et al., 2016). This leads us to hypothesize that HMZ might not be a “stable” MZ error in SA, but it might be context-dependent.

**Table 2:** Moderation of self-image on the effect of SA on self-referential HMZ.

	Self-referential HMZ	
	*B* (95% bCI)	*p*
*Moderation of Self-Esteem (n = 112)*		
SA (High vs. Low)	0.99 (−1.70 to 3.63)	0.37
Self-esteem	−0.25 (−0.42 to 0.06)	0.07
SA × Self-esteem	−0.16 (−0.58 to 0.29)	0.36
*Moderation of Negative Self-Schema (n = 109)*		
SA (High vs. Low)	1.77 (−0.57 to 4.15)	0.09
Negative Self-Schema	−0.04 (−0.08 to 0.16)	0.50
SA × Negative Self-Schema	−0.17 (−0.17 to 0.51)	0.21
*Moderation of Positive Self-Schema (n = 109)*		
SA (High vs. Low)	2.10 (−0.31 to 4.78)	0.45
Positive Self-Schema	−0.03 (−0.25 to 0.17)	0.81
SA × Positive Self-Schema	−0.21 (−0.46 to 0.79)	0.43

*Social anxiety (SA), Linear regression coefficients (B) of mean-centered predictors, 95% bootstrap confidence intervals (95% bCI), and p values (p).*

### The Context-Dependency of Hypermentalizing in Social Anxiety

The current findings point to the presence of HMZ associated with SA in a context-dependent manner. Specifically, it seems to depend on how arousing the social situation is. Given that the MASC is a procedure that places participants in a quiet situation (i.e., they are alone in a lab thinking about the mental states of the characters of a film, so they are not involved in the social situation that they evaluate), it is expected that SA will not be present, so it will not interfere with MZ activity. By contrast, given that in the MIMS participants are directly involved in a self-referential situation (an interview about themselves with a non-familiar adult), which is considered arousing for socially anxious people, SA is expected to be activated and therefore to impair MZ activity leading to HMZ. The current results support this hypothesis. HMZ was associated to SA only when it was self-referential, that is, while in the interview, but not while watching a film, as it happens when the MASC is used. This finding is important for several reasons. First, because it helps to clarify why previous studies using only the MASC (Hezel and McNally, 2014; Washburn et al., 2016) did not find a clear relationship between SA and general HMZ. Second, if the MZ error most expectably associated with SA is not stable but is context-dependent, then this suggests that people with SA can show adequate MZ capacity, which can be used in treatment. In light of these results, situational states of MZ merit as much attention as the general MZ style. These results suggest that considering both trait- and state- perspectives might shed new light in the study of MZ and mental disorders.

### Implications of Self-Referential Hypermentalizing for Treatment of Social Anxiety

Knowing the reluctance of people with SA to seek for help (American Psychiatric Association [APA], 2013), as well as their expectable difficulties to interact with a strange (the therapist), it is attractive to speculate implications for treatment of current results. The situational dependence of MZ errors highlights the existence of adequate MZ, which can be used for prevention and treatment. In this sense, the adaptation of Mentalization Based Treatment (MBT) (Bateman and Fonagy, 2008, 2009) to SA could be useful to: (a) focus on this Higher Order Cognition (MZ) involved in SA by definition (American Psychiatric Association [APA], 2013), (b) specifically increasing awareness of the situational tendency to HMZ, and (c) therefore inoculating against “delusional” beliefs about others’ intentions through introducing doubt of those distorted thoughts that have been held to be true (see Sharp et al., 2013).

Additionally, we should expect not only that HMZ might intervene in the rejection and delay of treatment of SA, but that projections and distortions derived from HMZ should be expected also in the therapeutic relationship. This is an additional reason to adapt MBT to SA, since MBT allows one to mentalize the therapeutic relationship and to address MZ errors *en vivo* in an experiential way. Moreover, MZ can be used even beyond MBT since it is present as a common active ingredient of treatments known to work (Wells and Purdon, 1999; Ahn and Wampold, 2001; Allen et al., 2008; Wampold, 2015; Hayes and Hofmann, 2017). Beyond the specific therapeutic program, MZ activity in treatment contributes strengthening of the therapeutic relationship (Allen and Munich, 2003; Wampold and Imel, 2015), reinforces trust in treatment and in the clinician (Fonagy and Allison, 2014; Fonagy et al., 2015), and therefore, fosters adherence that further increases probability of success (Sandi-Urena et al., 2011; Tschuschke et al., 2015). So, given the social dimension of SA, and therefore the importance of social cognition, mentalization should be active ingredient in treatment, both as a method (using adequate MZ to foster recovery), and an objective (to detect, understand and deactivate context-dependent HMZ).

### Explicit Self-Image Does Not Moderate Self-Referential Hypermentalizing

Our second question considered the role of self-image in context-dependent HMZ. It was hypothesized that a negative self-image might be involved in the excessive suspiciousness of people with SA, thus leading them to expect the same negative judgment from others that they have toward themselves, and therefore to over-attribute the intention of scrutiny and negative judgment (HMZ). Current results do not support this idea. Nor self-esteem neither self-schemas moderate the association between SA and HMZ. This goes against both theoretical framework (Asendorpf et al., 2002; Wilson and Rapee, 2006; Zhao et al., 2012) and clinical experience, suggesting an important role of self-image in SA. One possible explanation is that the role of self-esteem in SA is different than the relationship analyzed here. It is possible that self-esteem does not moderate the association between SA and HMZ, but it contributes to general expectances of negative evaluation (i.e., a negative idea of others), or to specific ones (i.e., increasing the fear to possible negative evaluation before the interview). Moreover, self-esteem may interact with specific types or components of SA (e.g., general fear to negative evaluation), or with other factors (e.g., necessity of recognition or external validation), in the prediction of HMZ.

### Limitations and Guidelines for Further Research

However, it is also possible that implicit but not explicit self-esteem is moderating the association between SA and HMZ. All measures of self-esteem in the current study are based in self-reports. The lack of the implicit dimension is a limitation. In the field of psychosis, several studies go beyond the analysis of “simple” self-esteem to analyze the discrepancy between implicit and explicit self-esteem and its role in the development of paranoid delusion (Valiente et al., 2011, 2014). Further analyzes may consider whether implicit self-esteem or a combination of implicit-explicit are involved in the activation of context-dependent HMZ in SA.

Another limitation of the current study is that the sample, based on volunteers and predominantly including females, is not representative of the general population. In fact, given the over-representation of females (85%), conclusions may not be safely extrapolated to males. Therefore, replications with more representative samples are required. Future studies should also analyze the influence of general MZ profile in context-dependent MZ errors, as well as the relationship of self-esteem and MZ problems with different dimensions of SA (e.g., SA associated with interaction versus cognitive SA; physiological components versus behavioral components).

## Conclusion

Despite limitations, this study contains an important strength. To our knowledge, it is the first to use a measure of self-referential MZ activity induced in a live situation, and it is the first to show that MZ errors associated with a psychopathological condition can be context-dependent, and not necessarily a stable cognitive style. This highlights the importance of analyzing MZ from both trait- and state-points of view, because beyond the relatively stable social cognitive style, there can also be persistent context-dependent variations of MZ activity. If future studies support current findings, to identify why HMZ is activated in specific contexts, and therefore to elucidate which are the specific mechanisms that lead to HMZ might help to improve treatment of SA. This study highlights the underestimated role of social cognition in SA, and more specifically the context-dependency of HMZ in SA. Implications for treatment are also important. Since HMZ is present in self-referential social situations and the therapeutic context is one of those, HMZ might be present in the therapeutic relationship and the clinician might be able to observe and directly treat one of the most essential symptoms of the SA spectrum. Future research should analyze whether treating HMZ in the therapeutic relationship improves cost-efficiency of current treatments and long-term benefits for people with SA.

## Data Availability

All datasets generated for this study are included in the manuscript and/or the Supplementary Files.

## Ethics Statement

The study meets ethical standards according to Declaration of Helsinki and was approved by the Ethics Committee of the Universitat Autònoma de Barcelona (CEEAH, Spain; CEEAH no. 4061). We report how we determined and obtained our sample, all data exclusions, all manipulations, and all measures in the study.

## Author Contributions

SB conceptualized and investigated the study and wrote the manuscript. SB, JV, and AT contributed to data curation and visualization. JV performed formal analysis. NB-V and SB acquired funding and provided resources. SB, JV, and CS defined the methodology. CS and NB-V validated the data. CS, NB-V, JV, and SB critically reviewed the manuscript.

## Conflict of Interest Statement

The authors declare that the research was conducted in the absence of any commercial or financial relationships that could be construed as a potential conflict of interest.

## Supplementary Material

DATA SHEET S1 Data used in the analyses.

## Abbreviations

BPD: borderline personality disorder
HMZ: hypermentalizing
MASC: movie for the assessment of social cognition
MIMS: method for inducing mentalization in a self-referential situation
MZ: mentalizing
SA: social anxiety
SAD: social anxiety disorder
ToM: theory of mind.
